# Open *versus* laparoscopic incisional hernia repair: nationwide database study

**DOI:** 10.1093/bjsopen/zraa010

**Published:** 2021-01-15

**Authors:** N A Henriksen, H Friis-Andersen, L N Jorgensen, F Helgstrand

**Affiliations:** 1 Department of Surgery, Zealand University Hospital, Koege, Denmark; 2 Department of Surgery, Regional Hospital, Horsens, Denmark; 3 Digestive Disease Centre, Bispebjerg Hospital, University of Copenhagen, Copenhagen, Denmark

## Abstract

**Background:**

Although laparoscopic repair of incisional hernias decreases the incidence of wound complications compared with open repair, there has been rising concern related to intraperitoneal mesh placement. The aim of this study was to examine outcomes after open or laparoscopic elective incisional hernia mesh repair on a nationwide basis.

**Methods:**

This study analysed merged data from the Danish Hernia Database and the National Patient Registry on perioperative information, 90-day readmission, 90-day reoperation for complication, and long-term operation for hernia recurrence among patients who underwent primary repair of an incisional hernia between 2007 and 2018.

**Results:**

A total of 3090 (57.5 per cent) and 2288 (42.5 per cent) patients had surgery by a laparoscopic and open approach respectively. The defect was closed in 865 of 3090 laparoscopic procedures (28.0 per cent). The median follow-up time was 4.0 (i.q.r. 1.8–6.8) years. Rates of readmission (502 of 3090 (16.2 per cent) *versus* 442 of 2288 (19.3 per cent); *P *=* *0.003) and reoperation for complication (216 of 3090 (7.0 per cent) *versus* 288 of 2288 (12.5 per cent); *P *<* *0.001) were significantly lower for laparoscopic than open repairs. Reoperation for bowel obstruction or bowel resection was twice as common after laparoscopic repair compared with open repair (20 of 3090 (0.6 per cent) *versus* 6 of 2288 (0.3 per cent); *P *=* *0.044). Patients were significantly less likely to undergo repair of recurrence following laparoscopic compared with open repair of defect widths 2–6 cm (*P *=* *0.002).

**Conclusion:**

Laparoscopic intraperitoneal mesh repair for incisional hernia should still be considered for fascial defects between 2 and 6 cm, because of decreased rates of early complications and repair of hernia recurrence compared with open repair.

## Introduction

Incisional hernia repair is a frequently performed surgical procedure, with variable complexity depending on the size and location of the hernia, previous operations, and patient co-morbidity^1^^,^^2^. Incisional hernias may be repaired by an open or a minimally invasive approach, but most minimally invasive repairs are performed by traditional laparoscopy, and in recent years some are being robot-assisted^3^. Traditionally, the laparoscopic technique requires placement of an intraperitoneal mesh (IPOM), which covers the defect and is fixated with tacks in a double-crown technique^4^^,^^5^. In an attempt to restore abdominal wall function and avoid bulging or seroma formation, suture closure of the defect before mesh placement has also been introduced (IPOM-Plus)^6^.

The advantage of laparoscopic repairs is shorter duration of hospital stay, faster recovery, and fewer wound complications. However, case series^7^^,^^8^ have shown that serious mesh-related complications may appear several years after the hernia repair, including small bowel obstruction due to adhesions to intraperitoneal mesh, and abscess or fistula formation owing to mesh erosion into bowel. These reports have led hernia surgeons to advocate mesh placement outside the peritoneal cavity. Furthermore, the possibility of tack fixation of the mesh causing chronic pain has led to the introduction of glue or fibrin fixation, or even no fixation^9^. Additionally, in recent decades, several new minimally invasive techniques have been presented. The advent of robotic surgery has made it easier to close the defect with sutures, and to place the mesh either preperitoneally or retromuscularly to avoid tack fixation^3^. Similarly, newer laparoscopic techniques such as the expanded-view totally extraperitoneal approach (eTEP) and the mini- or less-open sublay (MILOS) repair permit extraperitoneal mesh placement^10^^,^^11^. Although long-term outcomes are not yet available, these new techniques seem promising.

This study was undertaken to examine outcomes after open and laparoscopic incisional hernia repair (either with or without defect closure) on a nationwide basis with long-term follow-up. The aim was to compare open elective mesh repair and laparoscopic IPOM repair of primary incisional hernias with respect to rates of 90-day readmission, 90-day reoperation, and late operation for hernia recurrence. Whether these outcomes were associated with the hernia defect size was also assessed.

## Methods

Data on type of hernia, defect size, repair method, type of mesh, mesh size, and fixation method have been registered in the Danish Ventral Hernia Database since 2007 for all patients aged 18 years and above. In addition, all patients’ encounters with the Danish health system are registered in the National Patient Registry, which holds data on co-morbidity, emergency readmissions, and reoperations. Data from these two registries were merged by the use of each patient’s unique Danish identification number^12^. The study was approved by the Danish Data Protection Agency (REG-238-2018). Because the study relied on register data, no approval from an ethical committee was required. The study was approved and funded by the Danish Hernia Database.

### Inclusion criteria

All patients who underwent elective surgery on a primary incisional hernia by either an open or laparoscopic mesh technique between 1 January 2007 and 31 December 2018 were included. All types of elective laparoscopic repair were included irrespective of whether the defect was closed or not. If patients were registered with more than one incisional hernia repair during the inclusion period, the first procedure was defined as the index repair.

### Exclusion criteria

Patients were excluded if they underwent a primary incisional hernia repair with a Physiomesh™ product (Ethicon, Johnson & Johnson, Somerville, New Jersey, USA), irrespective of repair technique, as use of this mesh has been associated with a high recurrence rate resulting in its withdrawal from the market^13^^,^^14^. There were no exclusion criteria according to size, contamination status or presence of stoma.

### Variables

Age and sex were identified from the patient’s unique identification number. Co-morbidity associated with development of postoperative complications included diabetes and chronic obstructive pulmonary disease. The Charlson Co-morbidity Index score was recorded, categorized as none (0), mild (1), moderate (2) or severe (at least 3) co-morbidity^15^.

The type of incisional hernia was registered prospectively in the Danish Hernia Database, according to the direction of the previous laparotomy, as midline, transverse or other. Data on previous operations were not available. The maximum width and length of the fascial defect were recorded. Hernia size was based on the width of the defect, and grouped into small (0–2 cm), medium (more than 2 to 6 cm), large (more than 6 to 10 cm) and giant (over 10 cm). The repair method was registered as open or laparoscopic. Mesh placement was onlay, retrorectus, preperitoneal, intraperitoneal or other (including plug/bridging/inlay).

Readmission was registered as any emergency readmission to hospital within the first 90 days after the hernia repair. Readmission diagnoses were based on ICD-10 discharge codes^16^. Diagnoses were categorized as abdominal pain, superficial wound infection, deep wound infection, haematoma/postoperative bleeding, postoperative care and mobilization (including non-specific diagnoses such as incisional hernia, nausea, dizziness and fatigue), constipation, small bowel obstruction, cardiac disease, renal and urogenital disease, other gastrointestinal disease, pulmonary disease, neurological disease, hepatobiliary disease, skin disease, pain in the back or extremities, sepsis, and observation for an unspecified condition.

Reoperation was registered as an emergent surgical reintervention within the initial 90 days after the index incisional hernia repair. This outcome was classified into reoperation for surgical-site infection, reoperation for deep bleeding, diagnostic laparotomy or laparoscopy, surgery for small bowel obstruction or bowel resection, drainage of the abdominal cavity, endoscopy (gastroscopy/colonoscopy), pleural drainage or other.

The follow-up time was the interval between the primary surgery and the first operation for hernia recurrence, death, emigration, or last follow-up (31 December 2018). Hernia recurrence was defined by an operation for an incisional hernia recurrence registered in the Danish Ventral Hernia Database. Variables studied for the prediction of hernia recurrence included age in quartiles, sex, defect width, direction of the previous laparotomy incision (midline, transverse or other), use of mesh, surgical approach (open or laparoscopic), and readmission and reoperation within 90 days.

### Statistical analysis

Categorical and continuous data are presented as numbers with percentages and median (i.q.r.) respectively. Data were analysed using the χ^2^ or Mann–Whitney *U* test. Univariable and multivariable Cox regression analyses were undertaken to identify variables associated with operation for recurrence. Variables likely to be associated with operation for recurrence (*P *<* *0.200 in univariable analysis) were included in the multivariable analysis. The cumulative incidence of operation for hernia recurrence was analysed using survival tables, compared with log rank test, and illustrated by means of Kaplan–Meier plots. Two-tailed *P *<* *0.050 was considered statistically significant. The statistical analysis was done using SPSS^®^ (IBM, Armonk, New York, USA).

## Results

A total of 2288 (42.5 per cent) and 3090 (57.5 per cent) patients had surgery by an open and laparoscopic approach respectively. Patient demographics are shown in *Table 1*. The median duration of hospital stay was 1 (i.q.r. 0–2) day after laparoscopic repair, significantly shorter than the 2 (0–5) days after open repair (*P *=* *0.001). The overall rate of operation for recurrence was 7.6 per cent after open and 7.2 per cent after laparoscopic repairs.

**Table 1:** Demographics of 5378 patients who had surgery for primary incisional hernia by an open or laparoscopic approach

	Open (*n* = 2288)	Laparoscopic (*n* = 3090)
**Year of hernia repair**		
2007–2010	413 (18.1)	846 (27.4)
2011–2014	849 (37.1)	1159 (37.5)
2015–2018	1026 (44.8)	1085 (35.1)
**Age (years)** ^*^	61 (50–70)	61 (50–69)
**Sex ratio (F : M)**	1148 : 1140	1693 : 1397
**Charlson Co-morbidity Index score**		
0 (no co-morbidity)	1005 (43.9)	1520 (49.2)
1 (mild co-morbidity)	452 (19.8)	615 (19.9)
2 (moderate co-morbidity)	408 (17.8)	495 (16.0)
≥ 3 (severe co-morbidity)	423 (18.5)	460 (14.9)
		
**Type of hernia**		
Midline	1312 (57.3)	1494 (48.3)
Transverse	699 (30.6)	1281 (41.5)
Other	277 (12.1)	315 (10.2)
**Size of hernia (cm)** ^*^		
Width	5 (2.5–10)	5 (3–7)
Length	6 (3–14)	5 (3–10)
**Defect width (cm)**		
0–2	548 (24.0)	479 (15.5)
> 2–6	800 (35.0)	1674 (54.2)
> 6–10	522 (22.8)	641 (20.7)
> 10	418 (18.3)	296 (9.6)
**Closure of defect**	1407 (61.5)	865 (28.0)
**Mesh fixation**		
Sutures	1393 (60.9)	31 (1.0)
Tackers	156 (6.8)	2907 (94.1)
No fixation	347 (15.2)	10 (0.3)
Other	392 (17.1)	142 (4.6)
**Mesh location**		
Onlay	607 (26.5)	0 (0)
Retrorectus	1019 (44.5)	0 (0)
Preperitoneal	175 (7.6)	94 (3.0)
Intraperitoneal	386 (16.9)	2996 (97.0)
Other	101 (4.4)	0 (0)

Values in parentheses are percentages unless indicated otherwise;

* values are median (i.q.r.).

The 90-day rates of readmission (502 of 3090 (16.2 per cent) *versus* 442 of 2288 (19.3 per cent); *P *=* *0.003) and reoperation (216 of 3090 (7.0 per cent) *versus* 288 of 2288 (12.5 per cent); *P *<* *0.001) were significantly lower after laparoscopic than open repairs (*Table 2*). Readmission owing to surgical-site infection was significantly more frequent following open repair (58 of 2288 (2.5 per cent) *versus* 14 of 3090 (0.4 per cent); *P *<* *0.001). Readmission because of pain was significantly more common among patients operated on laparoscopically (102 of 3090 (3.3 per cent) *versus* 44 of 2088 (2.1 per cent); *P *=* *0.011). Reasons for emergency readmissions are shown in *Table 3*. Potentially life-threatening surgery for bowel obstruction or bowel resection was twice as common after laparoscopic compared with open repair (20 of 3090 (0.6 per cent) *versus* 6 of 2288 (0.3 per cent) respectively; *P *=* *0.044). Reasons for reoperation are shown in *Table 4*.

**Table 2:** Outcomes after open or laparoscopic primary incisional hernia repair

	Open (*n* = 2288)	Laparoscopic (*n* = 3090)	***P*** ^†^
Duration of hospital stay (days)^*^	2 (0–5)	1 (0–2)	< 0.001^‡^
90-day readmission	442 (19.3)	502 (16.2)	0.003
90-day reoperation	288 (12.6)	216 (7.0)	< 0.001
Operation for hernia recurrence	175 (7.6)	221 (7.2)	0.491
Death within 90 days	14 (0.6)	14 (0.5)	0.424
Follow-up (years)^*^	3.5 (1.5–6.1)	4.6 (2.2–7.3)	< 0.001^‡^

Values in parentheses are percentages unless indicated otherwise;

* values are median (i.q.r.).

† χ^2^ test, except

‡ Mann–Whitney *U* test.

**Table 3:** Reasons for 90-day readmission after open and laparoscopic incisional hernia repair

	Open (*n* = 442)	Laparoscopic (*n* = 502)
**Readmission directly linked to hernia repair**		
Abdominal pain	44 (10.0)	102 (20.3)
Superficial wound infection	58 (13.1)	14 (2.8)
Deep wound infection	9 (2.0)	10 (2.0)
Haematoma/postoperative bleeding	34 (7.7)	32 (6.4)
Postoperative care and rehabilitation	57 (12.9)	49 (9.8)
Constipation	7 (1.6)	17 (3.4)
Small bowel obstruction	10 (2.3)	22 (4.4)
**Readmission possibly linked to hernia repair**		
Cardiac disease	22 (5.0)	32 (6.4)
Renal and urogenital disease	23 (5.2)	20 (4.0)
Other gastrointestinal disease	16 (3.6)	13 (2.6)
Pulmonary disease	13 (2.9)	12 (2.4)
Neurological disease	12 (2.7)	15 (3.0)
Hepatobiliary disease	4 (0.9)	9 (1.8)
Skin disease	3 (0.6)	6 (1.2)
Pain in back or extremities	4 (0.9)	6 (1.2)
Sepsis	6 (1.4)	3 (0.6)
Observation for unknown condition	120 (27.1)	140 (27.9)

Values in parentheses are percentages.

**Table 4:** Reasons for 90-day reoperation after open and laparoscopic incisional hernia repair

	Open (*n* = 288)	Laparoscopic (*n* = 216)
Reoperation for superficial wound complication	152 (52.8)	42 (19.4)
Reoperation for deep infection/abscess	6 (2.1)	1 (0.5)
Reoperation for deep bleeding	19 (6.6)	11 (5.1)
Exploratory laparotomy	10 (3.5)	4 (1.9)
Laparoscopy	2 (0.7)	26 (12.0)
Surgery for bowel obstruction or bowel resection	6 (2.1)	20 (9.3)
Drainage of the abdominal cavity	10 (3.5)	11 (5.1)
Endoscopic procedure (gastroscopy/colonoscopy)	39 (13.5)	33 (15.3)
Pleural drainage	1 (0.3)	1 (0.5)
Other	43 (14.9)	67 (31.0)

Values in parentheses are percentages.

**Fig. 1:**
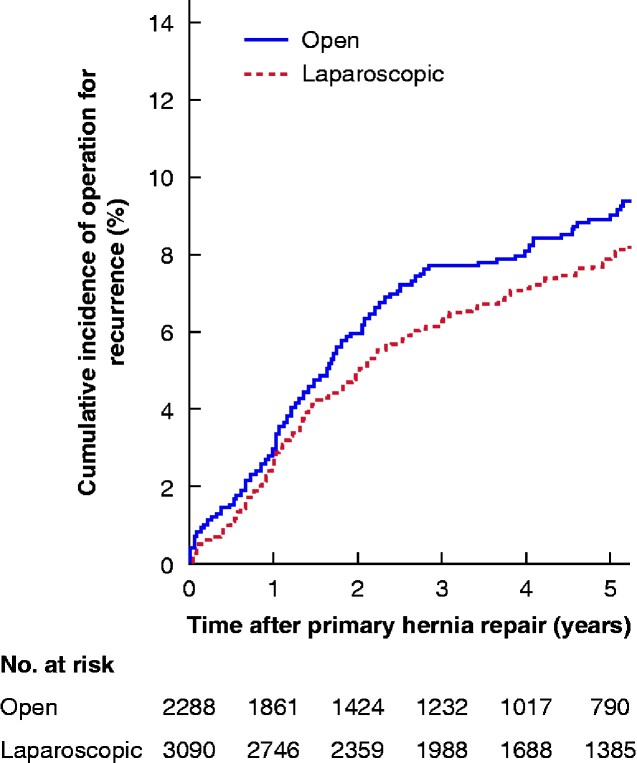
Cumulative incidence of operation for recurrence after primary elective incisional hernia repair with mesh by an open or laparoscopic approach, all hernia sizes *P* = 0.055 (log rank test).

The median follow-up time was 4.0 (1.8–6.8) years. The 4-year cumulative incidence of operation for hernia recurrence was 8.4 per cent after open and 7.4 per cent after laparoscopic repairs (*P *=* *0.055) (*Fig. 1*).

In a multivariable Cox regression analysis, age over 61 years, mild co-morbidity, laparoscopic repair, and defect closure were factors independently associated with a decreased risk of operation for hernia recurrence. Conversely, a defect width larger than 10 cm and reoperation within 90 days were factors independently associated with operation for hernia recurrence (*Table 5*).

**Table 5:** Univariable and multivariable Cox regression analyses of variables associated with operation for recurrence after primary incisional hernia repair

	Univariable analysis		Multivariable analysis	
	Hazard ratio	*P*	Hazard ratio	*P*
**Female sex**	0.91 (0.75, 1.11)	0.373		
**Age (years)**				
18–50	1.00 (reference)		1.00 (reference)	
51–61	0.87 (0.67, 1.13)	0.308	0.79 (0.60, 1.02)	0.079
62–70	0.83 (0.64, 1.08)	0.173	0.70 (0.54, 0.92)	0.012
71–103	0.78 (0.58, 1.04)	0.098	0.65 (0.48, 0.87)	0.005
**Charlson Co-morbidity Index score**				
0 (no comorbidity)	1.00 (reference)		1.00 (reference)	
1 (mild co-morbidity)	0.61 (0.46, 0.79)	0.001	0.58 (0.44, 0.76)	0.001
2 (moderate co-morbidity)	0.82 (0.61, 1.11)	0.192	0.81 (0.60, 1.09)	0.171
≥ 3 (severe co-morbidity)	0.76 (0.55, 1.06)	0.103	0.79 (0.57, 1.09)	0.156
**Defect width (cm)**				
0–2	1.00 (reference)		1.00 (reference)	
> 2–6	1.29 (0.95, 1.75)	0.098	1.31 (0.96, 1.77)	0.087
> 6–10	1.32 (0.95, 1.86)	0.098	1.27 (0.90, 1.78)	0.169
> 10	1.73 (1.22, 2.47)	0.002	1.53 (1.07, 2.19)	0.019
**Type of hernia**				
Midline	1.00 (reference)			
Transverse	0.99 (0.80, 1.22)	0.924		
Other	1.08 (0.78, 1.49)	0.652		
**Laparoscopic repair**	0.82 (0.68, 1.00)	0.055	0.76 (0.61, 0.93)	0.011
**Closure of hernia defect**	0.67 (0.53, 0.85)	0.001	0.60 (0.47, 0.76)	< 0.001
**Readmission within 90 days**	1.35 (1.07, 1.72)	0.012	1.07 (0.83, 1.38)	0.613
**Reoperation within 90 days**	1.98 (1.53, 2.59)	< 0.001	1.73 (1.30, 2.30)	< 0.001

Values in parentheses are 95 per cent confidence intervals.

**Fig. 2:**
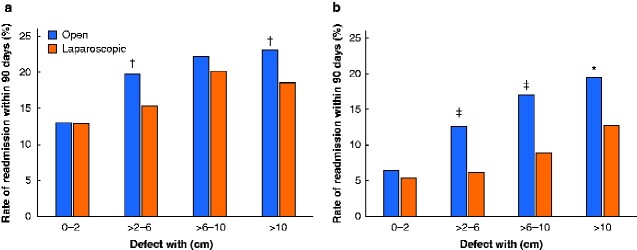
Ninety-day rates of readmission and reoperation after primary elective open or laparoscopic incisional hernia repair **a** Readmission and **b** reoperation. *P < 0.050, †*P* < 0.010, ‡*P* < 0.001 (χ^2^ test).

**Fig. 3:**
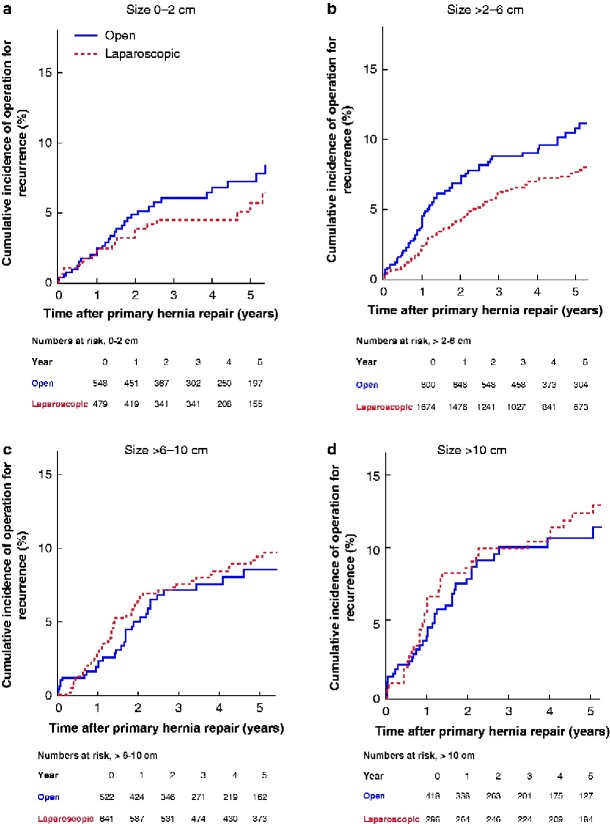
Cumulative incidence of operation for hernia recurrence after primary elective incisional hernia repair by an open or laparoscopic approach according to maximum width of fascial defect **a** 0–2 cm, **b** more than 2 to 6 cm, **c** more than 6 to 10 cm, and **d** over 10 cm. **a** *P* = 0.451, **b** *P* = 0.002, **c** *P* = 0.739, **d** *P* = 0.490 (log rank test).

Patients were grouped according to the maximum fascial hernia defect width; 1027 had small (0–2 cm), 2474 had medium-sized (more than 2 to 6 cm), 1163 had large (more than 6 to 10 cm), and 714 had giant (over 10 cm) hernias. A total of 1674 patients (67.6 per cent) with medium-sized incisional hernias underwent laparoscopic repair. Rates of 90-day readmission, 90-day reoperation and operation for hernia recurrence were significantly lower in this group than in the group that had to open repair (*Figs 2* and *3*). Patients undergoing laparoscopic repair of a large or giant incisional hernia had a significantly decreased rate of 90-day reoperation compared with those having open repair (*Fig. 2*), but without subsequent alteration in the risk of repair of a recurrent hernia (*Fig. 3*).

## Discussion

In this nationwide database study, traditional laparoscopic incisional hernia repair with intraperitoneal mesh fixated by tackers was associated with a shorter hospital stay as well as lower rates of early complications compared with open incisional hernia repair. Laparoscopic repair of medium-sized incisional hernias with defect widths between 2 and 6 cm was associated with lower rates of readmission, reoperation and operation for recurrence than the open approach. However, postoperative complications were more severe after laparoscopic repair.

The choice between the open or laparoscopic technique as the optimal approach for incisional hernia repair has been discussed for decades. In a Cochrane review^17^ from 2011 based on ten RCTs with a total of 880 patients, it was concluded that recurrence rates following open and laparoscopic surgery were comparable. Duration of hospital stay was shorter and the incidence of surgical-site infection was significantly lower in the laparoscopic group, whereas there were no significant differences in other complications. These findings were later confirmed by other reviews and meta-analyses^18–21^. In addition, one of the meta-analyses^18^ reported a higher rate of perioperative bowel injuries in the laparoscopic group. The main drawback of the RCTs included in the meta-analyses is a short follow-up time with a maximum of 2 years, which is why the hernia recurrence rates may have been underestimated.

In a recent large database study from the German Herniamed Registry^22^, a total of 9907 patients who had elective incisional hernia repair with 1-year follow-up were analysed. Laparoscopic IPOM repair was associated with a decreased rate of postoperative complications and reoperation for complications than open sublay repair, whereas the risk of intraoperative enterotomy was slightly increased during laparoscopic repair. Both of these findings are in line with the results of the present study. Two smaller recent studies^23^^,^^24^ also reported reduced rates of postoperative complications and shorter hospital stay after laparoscopic IPOM compared with open repair.

One concern about placing the mesh intraperitoneally is the risk of late mesh-related complications. In an American database study^7^, 733 patients undergoing laparoscopic ventral hernia repair were followed for a mean of 19 months for any subsequent abdominal operations. The overall rate of abdominal reoperation was 17 per cent, and most of the reoperations were performed safely. Rates of reoperation were 5 per cent for hernia recurrence, 2 per cent for bowel obstruction, and 2 per cent for mesh infection. The overall incidence of enterotomy or unplanned bowel resection at reoperation was 4 per cent. In another large case series^25^ of 1326 patients undergoing laparoscopic ventral hernia repair with defect closure and intraperitoneal mesh, both overall morbidity and hernia recurrence rates were low after a mean follow-up of 78 months. A second laparoscopy was undertaken in 126 patients, of whom 45 per cent had no adhesions, 42 per cent had minor adhesions, and the remainder had serosal adhesions^25^. In a large cohort study^8^ based on data from the Danish Hernia Database, the cumulative risk of mesh-related complications was lower after laparoscopic repairs than open repairs. Preceding placement of an intraperitoneal mesh was not associated with a significantly higher risk of complications compared with other mesh positions.

Even though the rates of short- and long-term complications after the laparoscopic approach were lower than those for open procedures, the possible complications may be more severe. For open repairs, the most frequent reason for readmission was superficial wound infection, and accordingly half of the 90-day reoperations were for wound infection. On the other hand, although the total frequencies were low, the rate of reoperations for severe complications was twice as high after laparoscopic procedures, possibly reflecting an increased risk of unidentified iatrogenic enterotomy at the initial hernia repair^18^. In recent years, there has been a trend towards open procedures with the aim of placing the mesh outside the abdominal cavity owing to the risk of long-term complications caused by interaction between the mesh and the bowel^7^^,^^25^. Novel minimally invasive procedures with extraperitoneal mesh placement appear as advantageous as the traditional laparoscopic technique regarding duration of hospital stay and surgical-site complications^11^^,^^26^. However, further studies are needed to clarify whether these new techniques are more promising in reducing rates of mesh complications and hernia recurrence than open or traditional laparoscopic IPOM repair.

The present study evaluated whether the size of the incisional hernia defects influenced postoperative outcomes. For patients with medium-sized hernias with defect widths between 2 and 6 cm, rates of readmission, reoperation, and reoperation for hernia recurrence were significantly decreased after a laparoscopic approach. For these patients, the laparoscopic repair technique seems advantageous. For patients with incisional hernia defects smaller than 2 cm, rates of readmission, reoperation, and recurrence were comparable between the groups. For patients with small incisional hernias, an open approach is feasible, and the risk of severe complications associated with the laparoscopic approach may not outweigh the short-term advantages. Although laparoscopic surgery is associated with a shorter hospital stay, the instrumental costs related to the use of coated meshes and tackers are higher. Therefore, for patients with the smallest hernia defects, laparoscopic repair should probably be reserved for those with a high risk of wound morbidity, such as current smokers and obese patients, and patients with more than one defect or defects outside the midline^27^. For patients with defect widths above 6 cm, the rate of early reoperation was decreased by the laparoscopic approach, but rates of readmission and repair for recurrence were comparable. In this group, it may be difficult to achieve both sutured defect closure and a mesh overlap of at least 5 cm, which is why laparoscopic repair can be challenging^6^^,^^28^. Defect width above 10 cm was an independent risk factor for recurrence. Patients with giant hernias are likely to benefit from referral to a specialized hernia centre, because of the need for a multimodal approach including preoperative optimization, and varying combinations of preoperative abdominal muscular paralysis with botulinum toxin A, minimally invasive techniques of component separation, and plastic surgery^2^. Such an initiative was taken among hernia surgeons in Denmark in 2010.

The present study is strengthened by the fact that it is based on nationwide data with a median follow-up of 4 years. Data from the Danish Hernia Database have been validated previously. The external validity of a nationwide database study is higher than that of RCTs with strict inclusion and exclusion criteria. However, there are limitations to this study. Patients were selected by the surgeon for either a laparoscopic or open approach based on certain unknown criteria, possibly leading to selection bias and unknown confounders in the results. Although the results presented suggest advantages and disadvantages for open and laparoscopic procedures, the findings do not necessarily apply to the individual patient. Data on BMI and smoking were not available for the entire cohort, and were thus not included in the analyses. As reported exclusively for patients who had surgery for hernia recurrence^29^, the true recurrence rate was underestimated. Furthermore, data were not available on long-term complications related to the hernia repair, such as small bowel obstruction, fistulas, and chronic mesh infections.

In the present study, laparoscopic incisional hernia repair was associated with a decreased rate of early complications and shorter hospital stay. For medium-sized hernias, the recurrence rate was also significantly decreased with a laparoscopic approach. The rising fear of placing mesh intraperitoneally may be justified by the fact that, even though laparoscopic IPOM is generally associated with a lower rate of short- and long-term complications, some are more severe possibly necessitating bowel resection. This should be considered when choosing the surgical procedure with the patient.

## Funding

Danish Hernia Database.
